# Perioperative Considerations of Novel Antidiabetic Agents in Heart Failure Patients Undergoing Cardiac Surgery

**DOI:** 10.3390/life15030427

**Published:** 2025-03-08

**Authors:** Ashley Wang, Savannah Bitzas, Dilsa Perez, Jonathon Schwartz, Saleem Zaidi, Jonathan Oster, Sergio D. Bergese

**Affiliations:** 1 Department of Anesthesiology, Stony Brook University Hospital, Stony Brook, NY 11794, USA; ashley.wang@stonybrookmedicine.edu (A.W.); jonathon.schwartz@stonybrookmedicine.edu (J.S.); saleem.zaidi@stonybrookmedicine.edu (S.Z.); jonathan.oster@stonybrookmedicine.edu (J.O.); 2 Renaissance School of Medicine, Stony Brook University, Stony Brook, NY 11794, USA; savannah.bitzas@stonybrookmedicine.edu (S.B.); dilsa.perez@stonybrookmedicine.edu (D.P.)

**Keywords:** diabetes mellitus, heart failure, GLP-1 receptor agonist, DPP-4 inhibitors, SGLT2 inhibitors, cardiac surgery, cardiovascular disease, glycemic control, perioperative management

## Abstract

Diabetes mellitus (DM) is a major risk factor for cardiovascular disease, including heart failure (HF). A high proportion of DM patients eventually require cardiac surgery. While the traditional approach to DM therapy focuses on tight glucose control with insulin and oral hypoglycemic agents, novel antidiabetic drugs have emerged over the past two decades that offer not only improved glycemic control but also cardiovascular and renal protection, such as benefits in HF management. The aim of this review is to examine and evaluate the perioperative risk and benefits of novel antidiabetic agents in HF treatment for both DM and non-DM patients undergoing cardiac surgery. We specifically studied glucagon-like peptide-1 receptor agonists (GLP-1RAs), dipeptidyl peptidase-4 (DPP-4) inhibitors, and sodium–glucose cotransporter 2 inhibitors (SGLT2is). Although studies on novel antidiabetic therapy in cardiac surgeries were limited, the results showed all three agents to be safe for use in the perioperative period, with SLGT2i demonstrating the most benefits in HF management for those with or without DM and kidney impairment undergoing cardiac surgery. Future research on larger study populations and using a more rigorous study design is necessary in bridging current knowledge to improve patient outcomes.

## 1. Introduction

Diabetes mellitus (DM) is a well-known risk factor for cardiovascular disease (CVD), which includes myocardial infarction (MI), strokes, and heart failure (HF). Among diabetics, CVD is one of the leading causes of mortality [1]. One hallmark of the DM-CVD interplay comes from the observation that the prevalence of HF in diabetics is between 9% to 22% in diabetic patients—four times higher than the general population [2]. Furthermore, among patients with HF, those with DM have a higher risk of hospitalization and worse health-related quality of life compared to those without [2]. As such, a comprehensive, multifactorial approach to the management of DM is essential to improved health outcomes in these patients.

**Figure 1:**
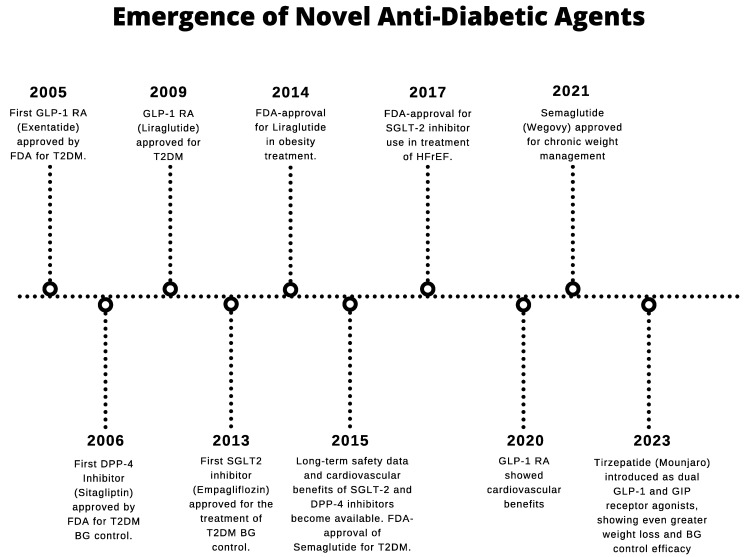
Timeline of emerging novel antidiabetic agents. GLP-1RA = Glucagon-like peptide-1 receptor agonists, FDA = Food and Drug Administration, T2DM = Type 2 diabetes mellitus, SGLT-2 = Sodium–glucose cotransporter 2, HFrEF = Heart failure with reduced ejection fraction, DPP-4 = Dipeptidyl peptidase-4, BG = Blood glucose, GIP = Glucose-dependent insulinotropic polypeptide.

Medical management for DM typically focuses on careful blood glucose (BG) control through insulin therapy, secretagogues, and oral hypoglycemic agents. However, this approach can be difficult to maintain given the challenges of frequent glucose monitoring, patient non-adherence, and adverse reactions from medications [3]. In addition, treating DM solely through tight blood sugar control does not adequately address the complexity of the disease across other organ systems such as the heart and kidneys. Given the need for new therapeutic approaches to DM management, several classes of novel antidiabetic agents have emerged over the past two decades for type 2 DM (T2DM) (Figure 1). Among these are glucagon-like peptide-1 (GLP-1) receptor agonists, dipeptidyl peptidase-4 (DPP-4) inhibitors, and sodium–glucose cotransporter 2 (SGLT-2) inhibitors. Besides providing improved BG control similar to traditional diabetic therapy, these novel agents have also demonstrated cardiovascular and renal protection, including benefits in HF management [3]. Approximately 20% of cardiac surgery patients have pre-existing diabetes mellitus, many of which require insulin therapy [4,5]. Because novel antidiabetic agents may confer organ protective effects separate from BG control, these agents may afford significant benefit in HF patients undergoing cardiac surgery, with or without DM.

**Figure 2:**
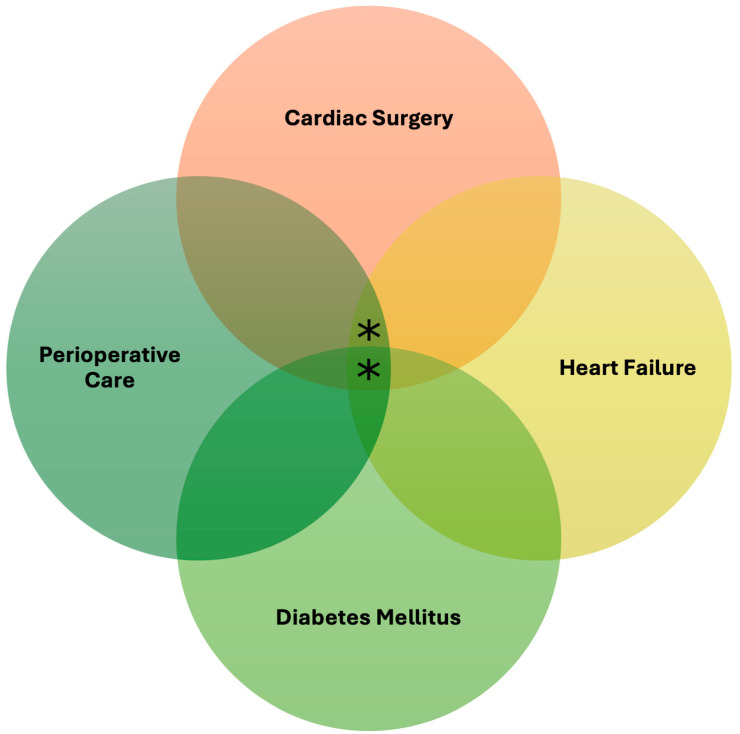
Venn diagram illustrating the various comorbidity and procedural features for the patient population of interest. The asterisks indicate the patient population of interest for the application of novel antidiabetic agents. Both heart failure and diabetes mellitus are common among cardiac surgery patients.

Every year, an estimated 1 to 1.5 million cardiac surgeries are performed globally [6]. In coronary artery bypass grafting (CABG), the prevalence of DM ranges from 12% to 40% [7,8,9,10]. Despite the rise in popularity of novel antidiabetic therapy for managing DM and HF, guidelines addressing perioperative use of these medications in cardiac surgery are limited. Since HF is associated with poorer health outcomes and higher mortality rates following cardiac surgery, novel antidiabetic agents may have a role in improving perioperative outcomes, independent of diabetic status [11,12]. Therefore, the aim of this review is to examine and evaluate the risk and benefits of novel antidiabetic agents in HF treatment for patients undergoing cardiac surgery, with or without DM (Figure 2). The scope of this paper includes the use of novel antidiabetic agents independently, not as combination therapies, in HF patients undergoing cardiac surgeries, including, but not limited to, CABG, valvular surgery, and durable mechanical circulatory support. This review will focus on outcomes for cardiac surgery patients in the immediate postoperative period, i.e., from the time a patient exits the operating room after surgery to when he or she approaches readiness for discharge.

## 2. Overview of Novel Antidiabetic Agents

### 2.1. Glucagon-like Peptide-1 Receptor Agonists (GLP-1RAs)

GLP-1 is a naturally occurring peptide produced from proglucagon within the intestinal mucosa, pancreatic islet alpha cells, and neurons in the nucleus of the solitary tract [13,14]. GLP-1 is an incretin hormone that regulates BG levels; it has a short duration of action as it is enzymatically degraded within 1 to 2 min by DPP-4. GLP-1 receptor is a G protein-coupled receptor with specific affinity for GLP-1 and is localized to the cellular membrane. GLP-1 receptor is involved in regulating insulin and glucagon secretion, maintaining glucose homeostasis [15,16]. GLP-1 receptor agonists (GLP-1RAs) are part of the new wave of antidiabetic agents that came to market, with Exenatide being the first GLP-1RA approved by the Food and Drug Administration (FDA) for T2DM in 2005 and Liraglutide approved by the FDA for obesity treatment in 2014. GLP-1RAs are GLP-1 analogs that stimulate insulin release and reduce glucagon production. GLP-1RAs, while structurally similar to GLP-1, include modifications to impede hydrolysis by DPP-4 to extend the half-life of the drug [15,16]. These GLP-1RAs have been shown to improve BG control in patients with T2DM and assist with weight loss in those experiencing obesity. This drug class should be considered in patients who have a contraindication to or intolerance of metformin, a glycated hemoglobin (HbA1c)—an indicator of long-term glycemic control—more than 1.5% over the target level, or those who cannot reach a target HbA1c within three months [17]. Minor side effects such as nausea, vomiting, and gastrointestinal (GI) upset have been reported [16]. Other effects include delayed gastric emptying and appetite suppression, which contribute to their success as a weight loss drug.

GLP-1RAs have shown a reduction in major adverse cardiac events (MACEs), all-cause mortality, and hospitalization for in the general population and those with T2DM [18,19]. These mortality benefits are evident in patients without baseline HF but absent in those with established HF [20].

Until recently, guidelines from the American Society of Anesthesiologists (ASA) recommended that GLP-1RAs be held on the day of surgery for those on a daily dosing schedule or the week prior to the surgery for those on a weekly dosing schedule, regardless of the indication for the drug (i.e., T2DM, weight loss) or the type of procedure/surgery [21]. This was based on the belief that withholding GLP-1RAs would lower the risk of delayed gastric emptying and aspiration. However, this approach did not fully account for the potential risks of stopping GLP-1RAs, including increasing blood sugar levels in DM patients [22]. As such, the ASA along with the American Gastroenterological Association, American Society for Metabolic and Bariatric Surgery, and others released an updated multisociety clinical practice guidance for the safe perioperative use of GLP-1RAs in October 2024 [23]. The new guidelines recommend that the use of GLP-1RAs in the perioperative period be based on shared decision-making of the patient with the procedural, anesthesia, and prescribing care teams. The guidance states GLP-1RA therapy may be continued preoperatively in patients without an elevated risk of delayed gastric emptying and aspiration [22,23].

On the other hand, GLP-1RA users considered at elevated risk include the following: (1) an escalation phase of GLP-1RAs—typically within 4 to 8 weeks of initiating therapy; (2) higher doses with a higher risk of GI side effects; (3) a weekly dosing schedule; (4) the presence of GI symptoms; and (5) medical conditions besides GLP-1RA usage, which may also delay gastric emptying, such as Parkinson’s, gastroparesis, and bowl dysmotility [22,23]. For these high-risk patients, the risks and benefits of withholding GLP-1RA must be assessed. If the decision is made to stop GLP-1RAs before a procedure, the medication should be held one day vs. one week based on GLP-1RA dosing schedule, following the original ASA guidelines. In addition, the updated guidance recommends preoperative diet modification to liquid diet for at least 24 h for patients at elevated risk. It also encourages the use of point-of-care gastric ultrasound on the day of the procedure if there is clinical concern for retained gastric content [22,23]. To decrease aspiration risk during the procedure, rapid sequence induction (RSI) for tracheal intubation should be considered for airway protection in patients with symptoms of delayed gastric emptying (i.e., nausea, vomiting, and bloating), inadequate fasting time, emergency surgery, or other comorbid conditions (i.e., obesity and DM) that put them at an increased risk of aspiration [24]. When there is clinical concern for retained gastric contents, the anesthesia provider should engage patients in shared decision making while weighing the benefits and risks of rapid sequence induction for tracheal intubation in general anesthesia to reduce aspiration risk vs. procedure cancellation.

### 2.2. Dipeptidyl Peptidase-4 (DPP-4) Inhibitors

The catalytic glycoprotein DPP-4 (dipeptidyl peptidase-4), also known as CD26, is expressed in various cell types throughout the body, including hepatocytes, endothelial cells, and islet endocrine cells [25,26]. Incretin hormones such as GLP-1 and glucose-dependent insulinotropic polypeptide (GIP) are rapidly metabolized by DPP-4 to inactive forms [27]. DPP-4 inhibitors were developed to prevent the degradation of GLP-1 and GIP, and thereby extend the duration that incretin hormones stimulate insulin secretion. Satagliptin was the first DPP-4 inhibitor in the market to be approved by the FDA in 2006, shortly after the European Medicines Agency approved vidagliptin. Between 2006 and 2013, three additional DPP-4 inhibitors—alogliptin, saxagliptin, and linagliptin—were approved by the FDA for the treatment of T2DM [25]. The DPP-4 inhibitors have been found to effectively improve glycemic control by reducing mean HbA1c by 0.5–0.8% [28]. When compared to sulfonylurea, DPP-4 inhibitors had lower rates of hypoglycemia and less weight gain [29]. Side effects associated with DPP-4 inhibitors include headache, upper respiratory tract infections, and musculoskeletal pains [28]. DPP-4 inhibitors join GLP-1RAs in the new wave of antidiabetic agents brought to market. When comparing DPP-4 inhibitors to GLP-1RAs, the superiority of GLP-1RAs was based on their ability to decrease HbA1c by 1–1.2%, their long half-life, and their ability to increase GLP-1 concentrations more than DPP-4 inhibitors, despite having more adverse effects [25,28]. DPP-4 inhibitors offer benefits over traditional antidiabetic agents like sulfonylureas, but seem less favorable compared to GLP-1RAs. Guidelines for the discontinuation of DPP-4 inhibitors before surgery vary, with some stating they should be withheld on the day of surgery while others allow their continuation [30,31].

### 2.3. Sodium–Glucose Cotransporter 2 Inhibitors (SGLT2is)

On average, the human kidneys filter 120 to 180 g of glucose from the body daily, with less than 0.5 g excreted in the urine in large part due to the role of SGLT-2 [32]. The main site of glucose resorption, SGLT-2s are found on the apical membrane of the proximal convoluted tubules to facilitate the transportation of glucose into the epithelia driven via the sodium electrochemical potential gradient across the membrane [32,33]. The first SGLT-2 inhibitor (SGLT2i), phlorizin, or phloretin-2′-*β*-D-glucopyranoside, is an organic compound initially extracted from apple tree bark and discovered by French chemists De Koninck and Stas in 1835 [34,35]. It took nearly a century for studies to report that the intravenous administration of phlorizin completely inhibited the reabsorption of renally filtered glucose in humans [36] and another half-century to show that diabetic rats treated with the compound showed increased glucose excretion through urine by preventing resorption, lowered BG levels, and restored insulin sensitivity [37]. These findings inspired further research to target SGLT2 as potential therapy for T2DM, eventually leading to the development of the first FDA-approved SGLT2i drugs, including canagliflozin, dapagliflozin, empagliflozin, and more [38].

While the current guidelines from the American Diabetic Association (2022) recommend metformin plus comprehensive lifestyle modification as a first-line treatment for T2DM, other therapies (GLP-1RAs and SGLT2is) may be appropriate, with or without metformin, for T2DM patients at a high risk of developing atherosclerotic cardiovascular disease (ASCVD), HF, and/or chronic kidney disease (CKD) based on an individual’s glycemic needs [39]. Whether used as a monotherapy or in combination with other glucose-lowering agents, SGLT2is have been shown to significantly lower HbA1c by 0.4 to 1.08% in several randomized controlled trials (RCTs) of 3 to over 8 months, allowing patients to achieve HbA1c targets and lower fasting plasma glucose [40]. Additionally, SGLT2is have been shown to promote weight loss and modestly reduce systolic blood pressure through osmotic diuretic effects [40].

SGLT2is are considered efficacious and safe, with mostly mild and manageable adverse effects. The most common are genital and urinary tract infections (UTIs), particularly yeast infections, from extensive glycosuria [33,35]. Other adverse effects include volume depletion from mild diuresis, hypoglycemia, and rarely but, most seriously, euglycemic diabetic ketoacidosis (DKA) [33]. Euglycemic DKA is characterized by inappropriately low insulin levels and elevated glucagon levels in the presence of serum glucose less than 250 mg/dL, leading to ketosis and severe metabolic acidosis [41]. Patients may be especially vulnerable to this potentially life-threatening situation in the perioperative setting, since surgical stress increases counter-regulatory hormones (e.g., glucagon, cortisol, and epinephrine) to promote a catabolic state, while SGLT2is also reduce serum glucose and indirectly inhibit insulin release [41]. Consensus statements based on expert opinion recommend stopping SGLT2is at least 24 h before elective surgeries or planned invasive procedures, though supporting evidence from clinical data is limited [41,42]. The FDA went a step further to change their recommendations, calling for cessation of SLT2i at least three days prior to surgery given the drug’s long half-life. Postoperatively, patients on SGLT2is should be observed closely for possible signs of adverse effects, continue insulin with dose adjustments made as required, and be monitored for acid/base status [41].

## 3. Novel Antidiabetic Agents in HF Management

Broadly speaking, heart failure (HF) is a complex condition characterized by the inability of the heart to pump blood effectively to meet the body’s metabolic demands [43]. Based on a 2021 proposal by major global scientific bodies, HF is more precisely defined as a clinical syndrome with symptoms and/or signs caused by a structural and/or functional cardiac abnormality and corroborated by elevated natriuretic peptide levels and/or objective evidence of pulmonary or systemic congestion [44]. Common symptoms that arise with HF include dyspnea, fatigue, peripheral edema, and decreased exercise capacity. A growing global pandemic, HF affects an estimate of over 64 million individuals worldwide as of 2017 [45]. In the USA, between 2015 and 2018, around 6.0 million Americans aged 20 or older suffered from HF [46]. The condition also poses a tremendous economic burden on the healthcare system of the country, costing an estimated USD 30.7 billion in 2012 and a projected cost of USD 69.8 billion by 2030 [45]. Risk factors for HF development include HTN, CAD, valvular heart disease, cardiomyopathies, and DM [43]. HF is typically classified by left ventricular ejection fraction (LVEF) and can be categorized into the following three groups: HF with reduced EF (HFrEF) for symptomatic HF with LVEF ≤ 40%, HF with mildly reduced EF (HFmrEF) for symptomatic HF with LVEF 41–49%, and HF with preserved EF (HFpEF) for symptomatic HF with LVEF ≥ 50% [44].

Research models show a downregulation of certain metabolic processes and an upregulation of stress responses and profibrotic pathways during HF [47]. Factors that determine the treatment of HF include its underlying cause, symptoms, and ejection fraction. Current treatment options aim to strengthen the heart, decrease heart rate, lower blood pressure, reduce fluid retention, and alleviate heart failure symptoms [48]. The 2022 Guideline-Directed Medical Therapy (GDMT) for HF, as outlined by the American Heart Association, American College of Cardiology, and the Heart Failure Society of America, includes four classes of medications used in HFrEF [49]: (1) renin–angiotensin system inhibition, using angiotensin receptor–neprilysin inhibitors, angiotensin-converting enzyme inhibitors, or angiotensin II receptor blockers; (2) beta-blockers; (3) mineralocorticoid receptor antagonists; and (4) sodium–glucose cotransporter-2 inhibitors (SGLT2is).

In a population-based retrospective cohort study, patients who underwent isolated primary CABG were found to have a higher prevalence of HFpEF in women [12]. This study also observed that both the operative and long-term mortality rates were higher in women with HFpEF [12]. At 30 days post-CABG, the proportion of deaths was highest in HF patients, specifically in those with HFrEF (5.1%; 244 of 4816 patients) [12]. Similarly, HFpEF was found to be highly associated with in-hospital mortality after CT surgery (hazard ratio [HR] 1.86, *p* = 0.01) [11]. Other outcomes, such as postoperative shock, occurred more frequently in HFpEF compared to control patients (17.8% vs. 6.7%, *p* < 0.001) [11]. HFpEF was also considered an independent risk factor of postoperative shock (adjusted odds ratio 2.9, *p* < 0.001) [11]. Overall, HF is a complex, multifactorial syndrome resulting in impaired ventricular filling and/or ejection [47,50]. The pathophysiology of HF involves signal transduction pathways affecting cardiomyocytes, fibroblasts, immune cells, microvascular endothelial cells, and lymphatic endothelial cells [51]. In HF, myocardial remodeling occurs in response to stress signals, which lead to cardiomyocyte hypertrophy or cell death [47,51].

### 3.1. GLP-1RAs in HF Management 

While the data for the use of GLP-1RAs in patients with HF are limited, research suggests that distinguishing between the type and chronicity of HF is important for assessing the potential benefits and harms of GLP-1RA use. In a meta-analysis performed by Ferreira et al., researchers found that GLP-1RAs reduced hospitalizations in patients with new onset HF, but not in those with HF at baseline [52]. Similarly, in the STEP-HFpEF trial, GLP-1RAs prevented hospitalization in patients with new onset-HF and reduced HF-associated symptoms in those with HFpEF [53]. HF patients receiving semaglutide showed greater improvement in exercise function and greater weight loss when compared to the placebo (−13.3% vs. −2.6% change in body weight after 1 year, respectively) [53]. Those in the semaglutide group had a significant reduction of 43.5% in the CRP level after 1 year compared to a 7.3% reduction in the placebo group [53]. These results may suggest an anti-inflammatory effect from GLP-1RA use in HF management. Most adverse events included GI upset, and semaglutide was associated with significantly less serious adverse events (death, arrhythmias, infection, etc.) when compared to the placebo [53]. Overall, there was a significant improvement in exercise function and weight loss in the semaglutide group compared to the placebo.

Alternatively, in the LIVE trial, GLP-1RAs resulted in either neutral or harmful effects in patients with known HFrEF with and without previously diagnosed T2DM [54]. While Liraglutide did not affect LV systolic function, it was significantly associated with an increased heart rate (six beats/min higher) and serious cardiac adverse events in HF patients when compared to the placebo [54]. Serious adverse events included death due to arrhythmia, aggravation of existing ischemic heart disease, and worsening of heart failure. Notedly, the group experiencing serious adverse events did not differ from the rest of the population when demographic and other health factors were accounted for [54].

In the EXSCEL (Exenatide Study of Cardiovascular Event Lowering) trial, there was no reduction in all-cause mortality in the HF subgroup (HR, 1.05), but mortality was reduced in the subgroup without HF (HR, 0.79, *p* = 0.03) [55]. Neves et al. performed a meta-analysis from the EXSCEL and FIGHT (Functional Impact of GLP-1 for Heart Failure Treatment) trials to investigate the risk of HF hospitalization with GLP-1RAs in patients with established HFrEF [56]. These authors found that GLP-1RAs increased the risk of HF hospitalizations in patients with an LVEF < 40%. The use of GLP-1RAs for other health conditions beyond T2DM has only recently been explored and more research is needed to identify the benefits and harms in patients with varying severity levels of HF.

### 3.2. DPP-4 Inhibitors in HF Management

After their FDA approval, four main clinical trials assessing the cardiovascular risk associated with DPP-4 inhibitors were conducted [25]. No significant difference in MACE, all-cause mortality, and heart failure was observed with DPP-4 inhibitors [25,57]. However, results from the SAVOR-TIMI 53 trial and the post hoc analysis of the EXAMINE trial indicated an increased hospitalization risk for heart failure associated with saxagliptin and alogliptin, respectively [57]. In 2016, the FDA issued a warning against two DPP-4 inhibitors (saxagliptin and alogliptin) regarding their role in increasing the risk of serious heart failure events. One possible mechanism to explain the worsening of heart failure in patients taking DPP-4 inhibitors is sympathetic overactivity through potentiation of endogenous stromal cell-derived factor 1, neuropeptide Y, and substance P [58]. These findings, however, are still controversial since subsequent studies have found mixed results with some studies stating no support has been found that DPP-4 inhibitors increase HF incidence [59].

As research continues to expand our understanding of these new antidiabetic agents, recent clinical guidelines have been modified by the American College of Physicians (ACP). The 2024 guidelines from the ACP recommended against the addition of DPP-4 inhibitors to metformin and lifestyle modification for the treatment of T2DM in patients with inadequate glycemic control to reduce morbidity and all-cause mortality [60]. Both GLP-1RAs and SGLT2is are preferred over DPP-4 inhibitors to reduce MACE, all-cause mortality, and heart failure in patients with T2DM based on the ACP guidelines. However, DPP-4 inhibitors have been found to provide some benefit in patients with diabetes and HFpEF. The use of DPP-4 inhibitors in DM patients with HFpEF, but not HFmrEF or HFrEF, was associated with a lower incidence of composite of cardiovascular death or HF hospitalization in these patients but not in HFmrEF or HFrEF [61]. Overall, DPP-4 inhibitors are less favored over the new antidiabetic agents (e.g., GLP-1RAs and SGLT2is), some benefits in HFmrEF are still limited by the data within the T2DM population.

### 3.3. SGLT2is in HF Management

A turning point for advancing treatment of cardiovascular complications in T2DM patients is the EMPA-REG OUTCOME trial (2015). A placebo-controlled RCT with over 7000 subjects, the landmark study was the first to show that a SGLT2i empagliflozin significantly lowered the risk of cardiovascular death (38% relative risk reduction) and HF hospitalization (35% relative risk reduction) for patients with T2DM and high cardiovascular risk [62]. The trial inspired a myriad of studies into SGLT2is as a potent agent for HF management in T2DM patients. The findings from these trials are summarized in Table 1.

A multinational double-blind, placebo-controlled RCT of over 17,000 patients, the DECLARE-TIMI 58 trial (2019) specifically looked at the effect of dapagliflozin in individuals with T2DM who have either established ASCVD (defined as clinically evident ischemic heart disease, ischemic cerebrovascular disease, or peripheral artery disease) or multiple risk factors for ASCVD (men age ≥ 55 and women age ≥ 60 with one or more traditional risk factors, including hypertension, dyslipidemia, or tobacco use) [63]. The study found that while dapagliflozin did not result in a higher or lower rate of MACE, it did lead to a lower rate of cardiovascular death or hospitalization for HF compared to placebo (4.9% for dapagliflozin vs. 5.8% for placebo). The advantage of SGLT2i use in T2DM patients for HF therapy was once again highlighted in the SOLOIST-WHF trial (2020), in which diabetic patients recently hospitalized for worsening HF were randomly assigned to receive sotagliflozin or placebo and followed up for 9 months [64]. The results showed that those started on sotagliflozin therapy before or shortly after discharge had a significantly lower total number of deaths from cardiovascular causes, as well as lower hospitalizations and urgent visits for HF (HR 0.67). Taken together, the remarkable cardiovascular benefits of SGLT2is, even in those without established CVD, allowed for the wider clinical application of the antidiabetic in HF management.

Subsequent RCTs on SGLT2is for HF treatment have specifically examined HFpEF and HFrEF patients regardless of DM status. Looking at subjects with HFpEF and a LVEF > 40%, the DELIVER trial (2022) demonstrated that dapagliflozin reduced the combined risk of worsening HF or cardiovascular death (16.4% in dapagliflozin vs. 19.5% in placebo) [65]. The EMPEROR-Preserved trial (2022) confirmed similar outcomes, noting a reduced rate of first hospitalization for HF or cardiovascular death in HFpEF patients with LVEF > 50% with or without T2DM. Furthermore, EMPEROR-Preserved also observed a slower rate of estimated glomerular filtration rate (GFR) decline in SGLT2i-treated HFpEF patients; this effect appears more pronounced in patients with DM [66]. These benefits are also extended to HFrEF patients with LVEF ≤ 40%, as demonstrated in the DAPA-HF (2019) and EMPEROR-Reduced (2022) trials. The DAPA-HF trial showed a lower risk of worsening HF (defined as hospitalization or urgent visit resulting in IV therapy for HF) or death from cardiovascular causes in the dapagliflozin group (16.3%) compared to placebo (21.2%) [67], whereas the EMPEROR-Reduced study found a reduced risk and total number of inpatients and outpatient worsening HF events, with early and sustained duration of effects [68]. Finally, the EMPULSE trial (2022) found that clinical benefits, along with improved symptoms, physical limitations, and quality of life, can be observed as early as 15 days and maintained through 90 days after initiating empagliflozin in patients hospitalized for acute HF, regardless of the degree of symptomatic impairment at baseline [69]. Overall, SGLT2i use in HF therapy appears beneficial across a wide range of HF severities and DM comorbidities.

## 4. Perioperative Considerations of Novel Antidiabetic Use in Cardiac Surgeries

A summary of the following studies presented on the use of antidiabetic agents in cardiac surgeries can be found in Table 2.

### 4.1. GLP-1RAs in Cardiac Surgeries 

The use of novel antidiabetic agents in patients undergoing cardiac surgery has increased due to the rise in popularity of these drugs in recent years. While GLP-1RAs have cardioprotective effects, its use in those undergoing cardiac surgery must also be explored as individuals on this drug often have cardiac-related comorbidities. Current research primarily compares the insulin requirements, BG levels, and glycemic variability of patients on GLP-1RAs to those on standard insulin who are undergoing cardiac surgery. Sindhvananda et al. found that the BG levels of those on Liraglutide with insulin were significantly lower than before, during, and after the operation as compared to insulin-only controls among patients with established T2DM undergoing cardiac surgery [70]. The perioperative mean difference of BG levels was 15.9 mg/dL, and those receiving Liraglutide with insulin had significantly less hyperglycemic episodes [70]. The study found that on postoperative day 0, the Liraglutide group had fewer patients who had BG > 180 mg/dL compared to the placebo (43.75% vs. 67.85%, *p* = 0.061). On postoperative day 2, the Liraglutide group demonstrated less glycemic variability compared to the placebo (SD 23.65 vs. 32.79 mg/dL, *p* = 0.018) [70].

These results are consistent with the findings of Hulst et al., who showed that those on Liraglutide required significantly lower total intraoperative insulin doses and a significantly lower number of insulin administrations compared to the placebo group. The mean intraoperative BG level in the Liraglutide group was 11.89 mg/dL lower than the placebo group [71]. Within the study, 16% of patients with previously diagnosed T2DM were evenly distributed between the intervention and placebo group, and, overall, these patients required more insulin and had higher perioperative BG levels; however, the results remained similar for those with or without T2DM and it did not result in a different effect on perioperative insulin. Postoperatively, the mean BG levels for the Liraglutide and placebo group were 8.8  ±  1.4 and 9.2  ±  1.4 (*p* = 0.006), respectively [71].

Makino et al. investigated the use of Liraglutide with insulin compared to insulin alone in patients with established T2DM undergoing cardiac surgery (2/3 CABG cases and 1/3 valve replacement cases), finding that the M values (indicative of proximity index of the target glucose level from day 1–10) were significantly lower in the Liraglutide group (5.8 vs. 12.3, respectively). These authors also found that the Liraglutide group required a significantly lower frequency of insulin dose modification and had a lower frequency of hypoglycemia [72]. Similarly, Oosterom-Eijmael et al. conducted a triple-blind randomized controlled trial comparing preoperative Liraglutide treatment to placebo in patients with or without established T2DM undergoing cardiac surgery, monitoring the effect on BG via Dexacom (continuous BG monitor); they found that Liraglutide increased the glycemic time in range by 72% compared to 47% in the control group [73]. These investigators also found that the peak mean glucose concentration occurred approximately 14–16 h postoperatively, with the Liraglutide group blood glucose peaking at approximately 8.2 mmol/L and the placebo group peaking at approximately 9.4 mmol/L [73].

Hulst et al. further explored the impact of Liraglutide on myocardial function after cardiac surgery in patients with or without previously diagnosed T2DM, finding that the effects on Liraglutide had lower intensive care unit (ICU) admittance [74]. They also found that despite left ventricular (LV) systolic function being comparable between the Liraglutide and placebo groups preoperatively, patients in the Liraglutide group had significantly higher rates of normal LV systolic function postoperatively [74]. While the Liraglutide group had a significantly higher heart rate (83 ± 11 beats/min) compared to the placebo group (77 ± 11 beats/min), there was no significant difference in mean arterial pressure or vasoactive/inotropic support needed throughout the postoperative course [74].

### 4.2. DPP-4 Inhibitors in Cardiac Surgeries 

An investigation into the use of DPP-4 inhibitors in patients undergoing cardiac surgery is necessary due to the increased use of these drugs in the recent decades and its controversial effects on heart failure. Research on DPP-4 inhibitors used in cardiac surgery is limited. In Cardona et al. [75], the use of sitagliptin in patients with T2DM undergoing CABG surgery was investigated for its ability to prevent and treat perioperative hyperglycemia. A total of 182 participants undergoing CABG were divided into two groups (sitagliptin or placebo). Patient characteristics included HbA1c 7.6% ± 1.5%. HF was not a clinical characteristic considered for this study. Sitagliptin or a placebo was given once daily, starting the day before surgery, and this continued until hospital discharge or for up to 10 days. The findings determined no differences in frequency of hypoglycemia or in mean daily glucose during the hospital stay, the duration of surgery, the duration of ICU or hospital length of stay, the need for vasopressors, perioperative complications, surgical reinterventions, or readmissions after hospital discharge. The study did find that lower insulin requirement was found in the sitagliptin group upon transfer from the ICU to the wards [75].

### 4.3. SGLT2is in Cardiac Surgeries 

Given the significant outcome improvement SGLT2i offers in HF management, it is possible that the novel antidiabetic agent may provide similar benefits in HF patients undergoing cardiac surgery. While limited, there have been studies exploring the perioperative use of SGLT2is in CABG and LVAD procedures. In a multi-center prospective cohort study, Sardu et al. recruited patients with ischemic heart disease (IHD) receiving CABG via minimally invasive extracorporeal circulation (MiECC) and examined the inflammatory burden (as measured by inflammatory markers such as leukocyte counts, CRP, IL-1, IL-6, and TNF-α), clinical events (namely any cause of death, cardiac death, non-fatal MI, stroke, and revascularization), and quality of life 5 years after surgery [76]. Out of the 648 subjects, 188 had preexisting T2DM, with 64 of those being SGLT2i users. The results found that patients without T2DM had a lower expression of inflammatory markers after the follow-up compared to those with T2DM. However, among T2DM patients, SGLT2i users had lower levels of inflammatory markers compared to the non-SGLT2i cohort. Similar trends were reflected in terms of clinical events; the non-SGLT2i cohort was found to have the highest rate of adverse outcomes compared to the non-T2DM and SGLT2i user cohorts at the 5-year follow-up [76]. Limitations of the study include the small sample size of the SGLT2i cohort, the lack of comparison with CABG via conventional extracorporeal circulation, and the lack of randomization in study design. But, overall, the study suggests that while T2DM might be a risk factor for worse prognosis in those receiving CABG via MiECC, treatment with SGLT2is for T2DM patients may confer some level of long-term anti-inflammatory protection to lower the rate of adverse clinical events. Of note, the article did not put focus on HF patients; rather, the authors recruited patients with IHD who qualify for CABG via MiECC.

Another prospective study by Al Namat et al. also investigated SGLT2i use in the setting of CABG surgeries, specifically assessing outcomes related to cardiac ischemia, glycemic status, and renal function at 6 months after surgery [77]. All patients were enrolled in a 6-month cardiac rehabilitation program with daily dapagliflozin treatment. The 120 subjects were split into four subgroups: T2DM patients with CKD, T2DM without CKD, prediabetes with CKD, and prediabetes without CKD. Of note, 87 out of the 120 patients (72.5%) were previously diagnosed with HF. The results showed an increase in mean EF by 8.43% across all patients, with more significant improvement in the prediabetes group compared to the T2DM group (10.14% vs. 6.98). For all patients, ischemic markers such as heart-type fatty-acid-binding protein (H-FABP) levels returned to normal at follow-up, while and troponin levels were significantly decreased (44,458 ng/L overall), particularly in CKD patients regardless of T2DM status (73,294 to 82,500 ng/L) [77]. Unsurprisingly, HbA1c levels improved in both the prediabetes and T2DM groups regardless of CKD status. Finally, there was an overall increase of 11.51 in the GFR for all patients, notably in the CKD with the T2DM cohort (18.93 increase) and the prediabetes group (14.89 increase) [77]. It should be noted that the study did not include a control group of patients not receiving SGLT2is given the largely established benefits of the drug in treating the pathologies. Other limitations include a disproportionately male study pool (less than 25% female) and the exclusion of CABG recipients with adequate glycemic control (i.e., without established diabetes or signs of prediabetes). Nevertheless, the analysis by Al Namat et al. demonstrated that regardless of one’s cardiac status (e.g., previous history of HF), treatment with SGLT2i dapagliflozin for CABG recipients significantly reduced ischemic risk, increased mean EF, enhanced glycemic status, and improved renal function across all patients regardless of T2DM and/or CKD status, which may prove especially beneficial for those with complex comorbidities. 

The number of studies assessing the safety and efficacy of SGLT2is in patients receiving durable mechanical support such as left ventricular assist devices (LVADs) is also growing. In a retrospective cohort study, Cagliostro et al. followed T2DM patients placed on LVAD support to examine the rates of SGLT2i use among the population, as well as subsequent changes in body mass index (BMI), HbA1c, diuretic dose (furosemide equivalent), and renal function over time. Out of the 509 T2DM patients on LVAD support, only 34 (6.7%) were treated with SGLT2is, and 17 of these patients (50%) had diagnosed HF characterized as ischemic cardiomyopathy (ICM) [78]. Cagliostro et al. reported that among the SGLT2i-treated cohort, there was no significant change in the BMI, HbA1c, or diuretic dose at 30-, 60-, and 180-day follow-ups. The only difference noted was a decrease in blood urea nitrogen (BUN) at 180 days. Potential SGLT2i-related adverse events such as genitourinary infections, acute kidney injury, limb amputations, and driveline infections occurred, though it is hard to know whether these were caused by SGLT2is or chance events given their nonspecific nature. No episodes of DKA, volume depletion, fracture, or hypersensitivity reactions were reported. Cagliostro et al. advocated for further research on the safety and impact of SGLT2is for LVAD patients [78].

In another retrospective study that also investigated SGLT2is in the setting of LVAD implantation, 29 out of 138 patients (21%) were started on SGLT2i therapy after LVAD placement, with a mean time of 108 days from LVAD placement to SGLT2i initiation [79]. Fardman et al. found a significant decrease in daily furosemide dose (23.5 mg/day) and weight loss (2.50 kg average weight loss) in patients started on SGLT2is. In addition, there was a significant reduction in systolic pulmonary artery pressure (sPAP, 5.6 mm Hg reduction) among a subgroup of 11 patients who underwent right heart catheterization during follow-up. Echocardiographic evaluation showed a higher prevalence of right ventricular (RV) dysfunction after SGLT2i initiation, though none of the patients with severe RV dysfunction required HF hospitalization during the study. Finally, there was no significant difference in LVAD parameters before or after SGLT2i initiation [79]. The study focused on outcome measures related to HF rather than DM and glycemic control (e.g., HbA1c, mean BG, and fasting BG). Overall, no SGLT2i-related adverse effects were reported. Although the study is limited in generalizability due to its small sample size and lack of comparison with a control group not treated with SGLT2is, it suggested that certain clinical benefits may come from initiating SGLT2is after LVAD placement.

## 5. Discussion and Future Directions

Our comprehensive review of the three classes of novel antidiabetic agents showed potential benefits in the perioperative setting for cardiac surgeries in HF patients, particularly for BG control and certain organ protective effects. These findings are summarized in Table 3. GLP-1RAs are safe for perioperative use and improve BG control, decrease insulin requirements, and support postoperative cardiac function. They do not increase the risk of hypoglycemic episodes or adverse effects when compared to insulin. Based on current guidelines, the case of whether or not GLP-1RAs should be stopped for surgery depends on clinical team’s judgment of patients’ aspiration risk. If a patient is deemed low-risk, GLP-1RAs may be continued until surgery. However, if the risk of aspiration is high and a decision is made to hold GLP-1RAs for surgery, the medication should be held on the day of surgery if the patient is on a daily dosing schedule or the week prior to surgery if the patient is on a weekly dosing schedule. Point-of-care gastric ultrasound can be used on the day of the procedure if there is clinical concern for retained gastric content [21,22,23]. After surgery, if the patient can safely tolerate oral intake, GLP-1RAs can be resumed the day after surgery if daily dosing or the next week if weekly dosing, although the current literature on when to resume this medication after surgery is limited. Within HF, GLP-1RAs reduce hospitalizations for new-onset HF and improves symptoms in HFpEF. However, GLP-1RAs generally do not improve health outcomes in patients with HFrEF and may even be harmful in those with a LVEF < 40% [52,56,58]. Overall, GLP-1RAs are beneficial in cardiovascular and renal outcomes within the general population and those in diabetes, but there are limited data available for these benefits within the HF population.

While studies on perioperative use of DPP-4 inhibitors in cardiac surgery are limited, they are generally safe without causing serious adverse effects, including hypoglycemia and weight gain, which are seen in DPP-4 inhibitor use [29]. The use of DPP-4 inhibitors led to lower mean daily insulin requirements in patients with T2DM undergoing CABG surgery [75]. However, saxagliptin and alogliptin were noted to increase hospitalization risk in HF patients [67], whereas DPP-4 inhibitors for HFpEF and DM were associated with lower rates of cardiovascular death or HF hospitalization [61]. Published recommendations on the discontinuation of DPP-4 inhibitors before surgery are less well established compared to those for SGLT2is and GLP-1RAs. In conclusion, DPP-4 inhibitors are generally safe but offer limited clinical benefits, with potential risks in heart failure patients and some benefit in HFpEF.

Out of the three novel antidiabetic agents discussed, SGLT2is showed the most promising perioperative benefits for cardiac surgeries. Not only does SGLT2is significantly lower HbA1c and fasting plasma glucose for T2DM patients, especially those at high risk for ASCVD, HF, and/or CKD, it also promotes weight loss and a reduction in high blood pressure [39,40]. Several RCTs established the remarkable benefits of SGLT2is in HF therapy, from a lower risk of worsening HF or cardiovascular deaths and slowing decline in kidney function to an improvement in symptoms, physical activity, and quality of life (Table 1). One study suggests that SGLT2is offer certain anti-inflammatory protective effects that reduce the rate of adverse clinical events in T2DM patients undergoing CABG via MiECC [76], while another demonstrated a lower ischemic risk, a higher mean EF, and an improved HbA1c, as well as renal function, in both T2DM and/or CKD patients undergoing CABG [77]. However, decisions on SGLT2i use in LVAD surgeries are mixed: one study showed no significant change in BMI, HbA1c, or diuretic dose but an increased risk of adverse effects (e.g., genitourinary infections, acute kidney injury, limb amputations, and driveline infections) [78], while the other noted a significant reduction in daily diuretic requirements, weight reduction, and sPAP reduction with no change in LVAD settings [79]. Based on current guidelines, stopping SGLT2is three to four days prior to cardiac surgery may be appropriate to minimize the risk of hypovolemia, hypoglycemia, and in extreme cases like euglycemia DKA. Only after careful postoperative monitoring of patients’ blood glucose and acid/base status, as well as ensuring adequate hydration and nutrition, should SGLT2is be resumed. In summary, SGLT2is provide the most advantages in HF management for those with or without T2DM and/or CKD in cardiac surgeries, as reflected in improved mean EF, reduced HbA1c, and lower inflammatory burden.

Given the many potential benefits that novel antidiabetic agents can offer in patients undergoing cardiac surgery, further research and studies are critical in bridging current knowledge and improving outcomes for all future patients. For one, most studies on the use of novel antidiabetic agents in cardiac surgeries presented in this review are small cohort studies with limited power. Larger study populations with a more rigorous study design would enhance validity and generalizability of findings. Also, a direct comparison of the three antidiabetic agents in cardiac surgeries proved to be challenging given that the studies presented highlighted different outcome measures. Future studies with consistent and standardized measures would allow for more rigorous comparison. While discussions on novel antidiabetic agents are focused on T2DM patients, there is room to investigate the use of these agents in those with type 1 DM. Additionally, investigation into combination therapy of two novel antidiabetic agents (such as GLP1-RAs and SGLT2is) may reveal new, potentially synergistic effects in treating patients with complex comorbidities. Finally, the majority of studies focused on the immediate postoperative period from the end of surgery to patient readiness to discharge. Further research on novel antidiabetic agents in the late postoperative period weeks, months, or even years after surgery would help guide chronic medication in future patients.

## 6. Conclusions

While studies on the use of novel antidiabetic agents in the setting of cardiac surgeries are limited at the moment, our review of the current literature suggests that all three agents (GLP-1RAs, DPP-4 inhibitors, and SGLT2is) are safe for use in the perioperative period, with SLGT2i showing the most benefits in HF management and other organ protection for those with or without DM undergoing cardiac surgery.

## Figures and Tables

**Table 1:** Clinical trials on SGLT2is for heart failure management in type 2 diabetes mellitus patients.

Clinical Trial	SGLT2i	Subjects	T2DM	HF	MACE	Target Population	Outcome
DECLARE-TIMI 58 (2018)	Dapagliflozin	17,160	+	↓	ND	Patients with T2DM and established atherosclerotic CV disease or multiple risk factors for CV disease	* SGLT2is did not result in a higher or lower rate of MACE, but did result in a lower rate of CV death or hospitalization for HF
DAPA-HF (2019)	Dapagliflozin	4744	−	↓	↓	NYHA II/III/IV HF and EF ≤40%	Lower risk of worsening HF or CV deaths, regardless of DM status
SOLOIST-WHF (2020)	Sotagliflozin	1222	+	↓	↓	T2DM patients recently hospitalized for worsening HF	Lower worsening HF and lower CV deaths
EMPEROR-Reduced (2020)	Empagliflozin	3730	−	↓	NA	NYHA II/III/IV HF and EF ≤ 40%	Lower risk and total number of inpatient and outpatient worsening HF at 12 days after starting SGLT2is
EMPULSE (2022)	Empagliflozin	530	−	NA	↓	Acute HF (both de novo and decompensated chronic HF with reduced or preserved EF, and with or without T2DM)	Improved symptoms, lesser physical limitations, and better quality of life as early as 15 days after starting SGLT2is and maintained through 90 days
DELIVER (2022)	Dapagliflozin	6263	−	↓	↓	Chronic HF and LVEF > 40%, i.e., preserved or mildly reduced	Lower combined risk of worsening HF or CV deaths
EMPEROR-Preserved (2022)	Empagliflozin	5988	+/−	↓	↓	Symptomatic HF and EF > 40%, elevated natriuretic peptide levels, and evidence of cardiac changes or previous HF hospitalization	Improvement in worsening HF outcomes; also slowed decline in kidney function—regardless of T2DM status

SGLT2i = Sodium–glucose cotransporter 2 inhibitor,T2DM = type 2 diabetes mellitus, + = patients with T2DM, − = patients without T2DM, HF = heart failure, ↓ = statistically significant reduction, MACE = major adverse cardiovascular event, NA = data not available, ND = no difference, CV = cardiovascular, NYHA = New York Heart Association functional classification, EF = Ejection fraction, LVEF = Left ventricular ejection fraction. * Worsening HF generally defined as hospitalization or urgent visits for HF.

**Table 2:** Summary of studies on the use of antidiabetic agents in cardiac surgeries.

Study: Author (Year), Type	Antidiabetic Agent	Subjects (n)	T2DM (n)	HF (n)	Others	Target Population and Cardiac Surgery Type	Outcomes
Sindhvananda et al. (2023) [70] RCT	GLP-1RA (Liraglutide)	56	Yes (56)	No		T2DM patients undergoing CABG	Significantly lower BG levels in patients on Liraglutide and insulin before, during, and after CABG compared to insulin-only controls.
Hulst et al. (2020) RCT [71]	GLP-1RA (Liraglutide)	278	Yes (43)	No		Patients with or without T2DM undergoing elective cardiac surgery	Significantly lower total intraoperative insulin doses and a significantly lower number of insulin administrations, as well as better postoperative BG control in the Liraglutide treatment group compared to the placebo control group.
Makino et al. (2019) [72] RCT	GLP-1RA (Liraglutide)	70	Yes (70)	No		T2DM patients undergoing CABG	Significantly lower M values (proximity index of target glucose level) in the Liraglutide treatment group compared to the insulin-only control group, suggestive of better glycemic control.
Oosterom-Eijmael et al. (2024) [73] Prospective cohort	GLP-1RA (Liraglutide)	25	Yes (3)	No		Patients with or without T2DM undergoing elective cardiac surgery	The Liraglutide treatment group had increased glycemic time in range compared to the placebo control group.
Hulst et al. (2020) [74] Secondary analysis	GLP-1RA (Liraglutide)	261	Yes (42)	No		Postoperative patients with or without T2DM after elective cardiac surgery	The Liraglutide group had a lower ICU admission rate and higher rates of normal LV systolic function postoperatively compared to the control group.
Cardona et al. (2021) [75] RCT	DPP-4 inhibitor (Sitagliptin)	182	Yes (182)	No		T2DM patients undergoing CABG	No differences in hypoglycemia rate, mean daily glucose, hospital stay, or readmissions after discharge in the DPP-4 inhibitor group compared to placebo. Lower insulin requirement in the DPP-4 inhibitor group upon transfer from ICU to wards.
Sardu et al. (2021) [76] Prospective cohort	SGLT2i	648	Yes (188)	No	Yes, IHD (188)	IHD patients undergoing CABG via MiECC	Patients without T2DM had lower levels of inflammatory markers postoperatively. Among T2DM patients, those treated with SGLT2is had lower levels of inflammatory markers compared to the non-SGLT2i group after surgery.
Al Namat et al. (2023) [77] Prospective cohort	SGLT2i (Dapagliflozin)	120	Yes (65)	Yes (87)	Yes, CKD (35)	Age ≥ 40 with clinical indication for CABG surgery	All patients underwent post-CABG rehabilitation and SGLT2i treatment. Regardless of cardiac statuses, rehabilitation and SGLT2i treatment led to improved mean EF, glycemic status, and renal function in patients with or without T2DM and with or without CKD.
Cagliostro et al. (2022) [78] Retrospective cohort	SGLT2i	34	Yes (34)	Yes (17)	Yes, CKD (16)	T2DM patients undergoing LVAD implantation	Lower BUN levels at 180-day follow-up with SGLT2i treatment but no significant change in BMI, HbA1c, or diuretic dose.
Fardman et al. (2023) [79] Retrospective cohort	SGTL2i	29	Yes (23)	Yes (29)		Patients undergoing LVAD implantation initiated on SGLT2i postoperatively	SGLT2i initiation after LVAD placement associated with decreased daily furosemide dose, weight, and sPAP but possibly higher RV dysfunction rate. No change in LVAD parameters.

T2DM = Type 2 diabetes mellitus, HF = Heart failure, RCT = Randomized controlled trial, GLP-1RA = Glucagon-like peptide-1 receptor agonist, CABG = Coronary artery bypass grafting, BG = Blood glucose, ICU = Intensive care unit, LV = Left ventricle, DPP-4 = Dipeptidyl peptidase-4, SGLT2i = Sodium-glucose cotransporter 2 inhibitor, IHD = Ischemic heart disease, MiECC = Minimally invasive extracorporeal circulation, EF = Ejection fraction, CKD = Chronic kidney disease, BUN = Blood urea nitrogen, LVAD = Left ventricular assist device, BMI = Body mass index, HbA1c = Glycated hemoglobin, sPAP = Systolic pulmonary artery pressure, RV = Right ventricule.

**Table 3:** Summary of novel antidiabetic agents and their effects.

	Antidiabetic Class		
	GLP-1RAs	DPP-4 Inhibitors	SGLT2is
Glycemic control	Significant reduction in HbA1c by ~1–1.2%.	Moderate reduction in HbA1c (0.5–0.8%).	Significant reduction in HbA1c (0.4–1.08%) and lower fasting plasma glucose as monotherapy or combined use with other agents.
HF with T2DM	Lower incidence of hospitalization for new-onset HFpEF but not in patients with established HF at baseline. Neutral or harmful in HFrEF.	Lower incidence of hospitalization and composite of cardiovascular death in HFpEF.	Improved outcomes (less worsening HF, hospitalization and/or urgent visits for HF, and quality of life) for HFpEF, HFmrEF, and HFrEF.
Cardiac surgery outcomes	Improved perioperative BG control with lower intraoperative insulin requirements.	Limited studies: No differences in frequency of hypoglycemia, mean daily glucose, hospital length of stay, surgical reinterventions, or readmissions after discharge in the DPP-4 inhibitor group compared to placebo. Lower insulin requirement in the DPP-4 inhibitor group upon transfer from ICU to wards.	CABG: Significant decrease in ischemic risk, improved mean EF, glycemic status, and renal function with or without T2DM, with or without CKD in the SGLT2i treatment group compared to the control. Lower-level inflammatory markers and ameliorated clinical outcomes at the 5-year postop point via MiECC. LVAD: Reduction in BUN and sPAP. Questionable reduction in weight/BMI and daily diuretic requirements. Possible higher RV dysfunction prevalence. No change in HbA1c or LVAD parameters.
Adverse events	GI upset (nausea/vomiting), delayed gastric emptying, appetite suppression, renal dysfunction, arrhythmias, and worsening of existing ischemic heart disease.	Upper respiratory tract infection, nasopharyngitis, headache, UTI, and arthralgia.	Volume depletion, UTI/yeast infection, hypoglycemia, and euglycemic DKA.
Perioperative recommendations	If the risk of delayed gastric emptying/aspiration is low, continue before surgery. If the risk of delayed gastric emptying/aspiration is high, hold GLP-1RAs on the day of surgery if on a daily dosing schedule or the week prior to surgery if on a weekly dosing schedule, as well as a 24 hr preoperative liquid diet Resume GLP-1RAs after surgery if patient is able to safely tolerate oral intake.	Continue DPP-4 inhibitor use or withhold on day of surgery. May resume DPP-4 inhibitor use immediately after surgery.	Hold SGLT2is at least 3 days before surgery. Monitor closely for glucose level and acid/base status after surgery and resume SGLT2is if able to tolerate oral intake and maintain adequate hydration.

GLP-1RA = Glucagon-like peptide-1 receptor agonist, DPP-4 = Dipeptidyl peptidase-4, SGLT2i = Sodium-glucose cotransporter 2 inhibitor, HbA1c = Glycated hemoglobin, HF = Heart failure, T2DM = Type 2 diabetes mellitus, HFpEF = Heart failure with preserved ejection fraction, HFrEF = Heart failure with reduced ejection fraction, HFmrEF = Heart failure with mildly reduced ejection fraction, BG = Blood glucose, ICU = Intensive care unit, CABG = Coronary artery bypass grafting, EF = Ejection fraction, CKD = Chronic kidney disease, MiECC = Minimally invasive extracorporeal circulation, LVAD = Left ventricular assist device, BUN = Blood urea nitrogen, sPAP = Systolic pulmonary artery pressure, BMI = Body mass index, RV = Right ventricle, GI = Gastrointestinal, UTI = Urinary tract infection, DKA = Diabetic ketoacidosis.
